# Edible Insects: Perceptions of Marketing, Economic, and Social Aspects among Citizens of Different Countries

**DOI:** 10.3390/foods12234229

**Published:** 2023-11-23

**Authors:** Raquel P. F. Guiné, Sofia G. Florença, Cristina A. Costa, Paula M. R. Correia, Luísa Cruz-Lopes, Bruno Esteves, Manuela Ferreira, Anabela Fragata, Ana P. Cardoso, Sofia Campos, Ofélia Anjos, Elena Bartkiene, Ilija Djekic, Irina M. Matran, Jelena Čulin, Dace Klava, Cristina Chuck-Hernández, Malgorzata Korzeniowska, Nada M. Boustani, Maria Papageorgiou, Bernardo Prieto Gutiérrez, Maša Černelič-Bizjak, Emel Damarli, Vanessa Ferreira

**Affiliations:** 1 CERNAS Research Centre, Polytechnic University of Viseu, 3504-510 Viseu, Portugal; sofiaflorenca@outlook.com (S.G.F.); amarocosta@sc.ipv.pt (C.A.C.); paulacorreia@esav.ipv.pt (P.M.R.C.); lvalente@estgv.ipv.pt (L.C.-L.); bruno@estgv.ipv.pt (B.E.); 2 Health Sciences Research Unit: Nursing (UICISA: E), Polytechnic University of Viseu, 3504-510 Viseu, Portugal; mmcferreira@gmail.com; 3 CIDEI-IPV Research Centre, Polytechnic University of Viseu, 3504-510 Viseu, Portugal; afragata@estgl.ipv.pt (A.F.); a.p.cardoso@esev.ipv.pt (A.P.C.); sofiamargaridacampos@gmail.com (S.C.); 4 CERNAS Research Centre, Polytechnic University of Castelo Branco, 6001-909 Castelo Branco, Portugal; ofelia@ipcb.pt; 5 Department of Food Safety and Quality, Lithuanian University of Health Sciences, 47181 Kaunas, Lithuania; elena.bartkiene@lsmuni.lt; 6 Department of Food Safety and Quality Management, Faculty of Agriculture, University of Belgrade, 11000 Belgrade, Serbia; idjekic@agrif.bg.ac.rs; 7 Department of Community Nutrition and Food Safety, GEP University MPhScTch of Targu Mures, 540139 Targu Mures, Romania; irina.matran@umfst.ro; 8 Maritime Department, University of Zadar, 23000 Zadar, Croatia; jculin@unizd.hr; 9 Faculty of Food Technology, Latvia University of Life Sciences and Technologies, LV 3001 Jelgava, Latvia; dace.klava@llu.lv; 10 Tecnologico de Monterrey, Institute for Obesity Research, Monterrey 64849, Mexico; cristina.chuck@tec.mx; 11 Faculty of Food Science, Wroclaw University of Environmental and Life Sciences, 51-630 Wrocław, Poland; malgorzata.korzeniowska@upwr.edu.pl; 12 Faculty of Business and Administration, Saint Joseph University, Beirut 1104 2020, Lebanon; nada.mallahboustany@usj.edu.lb; 13 Department of Food Science and Technology, International Hellenic University, 57001 Thessaloniki, Greece; mariapapage@ihu.gr; 14 BALAT Research Group, Faculty of Veterinary Medicine, University of León, 24071 León, Spain; bprig@unileon.es; 15 Department of Nutritional Counseling–Dietetics, Faculty of Health Science, University of Primorska, 6320 Izola, Slovenia; masa.cernelic@fvz.upr.si; 16 Research and Development Center, Altıparmak Food Coop., Çekmeköy, 34782 İstanbul, Turkey; emel.damarli@balparmak.com.tr; 17 Department of Nutrition, School of Nursing, UFMG—Federal University of Minas Gerais, Belo Horizonte 30130-100, Brazil; vanessa.nutr@gmail.com

**Keywords:** insect consumption, economy, social impact, questionnaire survey, marketing

## Abstract

Because edible insects (EI) have been, in recent years, recommended as a nutritious animal protein food with enormous environmental advantages over other sources of animal protein for human consumption, studies aimed at investigating the consumer perspective have become more prominent. Hence, this study intended to examine the perceptions of participants from different countries about the commercialization and economic and social impacts of edible insects. The study was made using a questionnaire survey, and data were collected in Brazil, Croatia, Greece, Latvia, Lebanon, Lithuania, Mexico, Poland, Portugal, Romania, Serbia, Slovenia, Spain, and Turkey. The final number of received answers was 7222 participants. For the treatment of the results, different statistical techniques were used: factor analysis, internal reliability by Cronbach’s alpha, cluster analysis, ANOVA to test differences between groups, and Chi-square tests. The results obtained confirmed the validity of the scale, constituted by 12 out of the 14 items initially considered, distributed by 4 factors: the first related to the economic impact of EIs, the second related to the motivation for consumption of EIs, the third related to the places of purchase of EIs, and the fourth corresponding to a question presented to the participants as a false statement. A cluster analysis allowed identifying three clusters, with significant differences between them according to all the sociodemographic variables tested. Also, it was found that the participants expressed an exceptionally high level of agreement with aspects such as the difficulty in finding EIs on sale, knowledge acting as a strong motivator for EI consumption, and the role of personalities and influencers in increasing the will to consume EIs. Finally, practically all sociodemographic variables were found to be significantly associated with perceptions (country, sex, education, living environment, and income), but not age. In conclusion, the perceptions about EI commercialization were investigated and revealed differences among samples originating from different countries. Moreover, the sociodemographic characteristics of the participants were found to be strongly associated with their perceptions.

## 1. Introduction

The United Nations (UN) estimates a continuing rise in the world population, which is a high challenge for sustainable development. The UN released in 2015 the Sustainable Development Goals (SDG) as a global movement to end poverty, protect the planet, and ensure that, by 2030, all people enjoy peace and prosperity [1].

Food security is at risk, and there is rising apprehension regarding the availability of the food required to meet the needs of the growing population [2,3]. Food production impacts the planet’s limited resources, such as farmland and freshwater, both when it comes to the production of vegetable or animal foods [4,5,6,7,8,9], but fisheries also have a huge impact on aquatic ecosystems [10,11]. Climate change also poses serious risks to the sustainable development of the planet and for food production. Factors such as lack of water, extreme weather conditions, droughts, floods, fires impacting forests and agricultural fields, and insect pests infecting agricultural crops cause important annual losses of food and are expected to be more severe in the future [12,13]. Therefore, high motivation has been observed globally among not only the scientific community, but also society in general and some political organizations to minimise the impacts of global warming and unsustainable development [1,14,15,16].

Alternative food sources, providing high nutritive value with more efficient use of resources, are a trend that aims to help sustainable development and efficient food providence. Edible insects (EI), among other sustainable trends like cultured meat or algae-based foods, constitute a way to meet these challenges, providing high protein contents more sustainably compared to conventional sources of protein for human consumption, like beef or pork protein [17,18]. For this reason, EIs have been suggested by the FAO—Food and Agriculture Organization of the United Nations [19] as part of a global strategy to achieve sustainable food security. 

Although insects have been part of the cultural culinary traditions among entomophagic communities in some parts of the world, their acceptance by consumers in most Western countries is faced with reluctance, suspicion, and even disgust or neophobia. Owing to these differences, the consumption and commercialization of EIs or insect-based foods are highly variable according not only to geographic factors, but also to other types of motivation [20,21,22]. Owing to these differences in food patterns among countries, the production and commercialization of insects are also very different among countries. While in some countries, insects are easily sold at all commercialization points, from street markets to other shops, in European countries, only some insects are allowed to be commercialized, as approved by the European Food Safety Authority (EFSA) under the Novel Food Regulation, and they are available in fewer selling points [23]. In the European Markets, edible insects and their derived ingredients are allowed to be marketed only after they have been authorized. This marketing authorisation is provided by a number of agents involved, namely the European Commission (EC), the safety evaluation of the novel food by the European Food Safety Authority (EFSA), and a favourable vote given by the EU Member States (MS) [24]. Under these restrictions, the farming of edible insects in European countries is also limited by EU regulations [25]. In contrast, in countries where entomophagy is part of the traditional food culture, the production and commercialization of edible insects are facilitated. The commercialization of edible insects was presented as a livelihood opportunity for local communities in the Lake Victoria basin [26]. The species that are produced and marketed also differ according to culture. For example, grasshoppers and small hemipterans known as ‘jumiles’ have been reported as the two main insects sold in the markets of the state of Morelos, Mexico [27].

Because edible insects’ consumption is highly variable among countries, and as a consequence, their commercialization is also differentiated, it is of interest to investigate how edible insects are marketed in different social–cultural environments, as well as to understand the role of consumers in these aspects. Hence, the aim of this study was to investigate the perceptions of participants from different countries about the commercialization and economic and social impacts of edible insects. This could be of particular importance to better understand how markets function and adapt selling strategies to increase consumers’ buying intentions. Also, in the ambit of this study, possible differences according to sociodemographic variables, such as country, sex, age, education, living environment, and income, were analysed. Finally, the scale items were analysed using a factor analysis to assess consistency.

## 2. Materials and Methods

### 2.1. Instrument and Data Collection

The data used in this study were collected by employing a questionnaire survey implemented by the team of the project EiSuFood, which was designed to investigate consumer issues related to EIs [28]. The Ethics Committee of the Polytechnic University of Viseu approved this questionnaire survey with reference 45/SUB/2021, and all ethical principles were strictly followed when designing the questionnaire and collecting the data, including those of the Declaration of Helsinki. Only adult citizens (18 to 88 years) who had expressed their informed consent were allowed access to the questionnaire to participate in the survey, and they were allowed to quit at any time without sending the answers if they wanted to. The questionnaire was answered online (on the Google Docs platform) and the invitation was made through an internet link shared by email or social media.

Table A1 in Appendix A presents the 14 questions included in the questionnaire that focused on the topics addressed in this particular study, namely two dimensions, Economic and Social aspects and Commercialization and Marketing. To measure the participants’ perceptions, they were asked to express their agreement with each item on a central five-point Likert scale as follows: 1 = strongly disagree, 2 = disagree, 3 = no opinion, 4 = agree, and 5 = strongly agree [29]. The questionnaire was prepared in the ambit of the EISuFood Project and validated through Structural Equation Modelling [28].

Although it is a convenience sample, some hint of the possible minimum sample size was calculated as indicative. For this, some assumptions were considered: Targeting 50% of the adult population; Confidence interval = 90%; Z score = 1.645; Power of the test = 5% (minimum acceptable probability of preventing type II error = 0.05) [30,31]. Based on these assumptions, for an infinite population, the number of responses stabilizes at 271. So, considering all countries involved, even for those with the highest population, a minimum of 271 participants should be guaranteed [32,33].

The data collection was carried out in 14 countries (Brazil, Croatia, Greece, Latvia, Lebanon, Lithuania, Mexico, Poland, Portugal, Romania, Serbia, Slovenia, Spain, and Turkey) in the second semester of the year 2021, and a total of 7222 participations were recorded. The initial target in each country was to obtain a minimum of 300 responses. 

### 2.2. Data Analysis

To evaluate the scale items, a Factor Analysis (FA) with the extraction methods of a Principal Components Analysis and Varimax rotation were applied. To fix the number of components to extract, the criterion used was Eigenvalues greater than 1. The Kaiser–Meyer–Olkin (KMO) measure to assess the adequacy of the sample was computed. Also the, Bartlett’s test was made to analyse the correlations between the variables studied [34]. For reference, values of KMO over 0.5 are acceptable, and the higher they are, the more suited the data are for the use of FA techniques [35]. Factor loadings with absolute values lower than 0.4 were excluded [36,37]. Cronbach’s alpha (α) was used to determine the internal consistency of each of the factors extracted [34,38]. The reference values for alpha considered were: acceptable consistency—0.5 < α < 0.7, good consistency—0.7 ≤ α < 0.8, and very good consistency—0.8 ≤ α [39,40,41]. 

Complementing FA, a cluster analysis was also performed based on the factors obtained through FA. For this, hierarchical clustering was applied with the Ward method and considering the Squared Euclidean distance to measure the intervals. For hierarchical clustering, the variables used were the four factors, and the results of the coefficients in the agglomeration schedule allowed fixing the number of clusters in three. Then, the same Ward method was run again but with fixing the number of clusters in three, and the solution was saved in the database for a posterior analysis of the clusters.

To evaluate differences between groups, ANOVA—analysis of variance was used, coupled with the post hoc test of Tukey, to identify differences between the means among groups. Also, chi-square tests were utilized when analysing differences between clusters based on sociodemographic variables. The coefficients of Cramer’s V were considered to evaluate the strength of the associations between the categorical variables tested. The reference values considered for the Cramer’s V coefficient were: V ≈ 0.1—weak association, V ≈ 0.3—moderate association, and V ≈ 0.5 or more—strong association [42].

The level of significance considered was 5%, and the software used in all statistical analyses was IBM^®^ SPSS Statistics software (Version 28). 

## 3. Results

### 3.1. Sample Characterization

**Figure 1:**
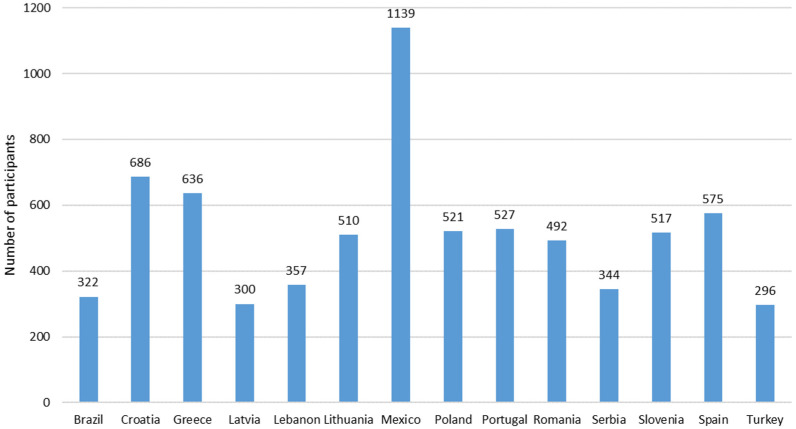
Distributions of the participants by country (*N* = 7222).

Figure 1 presents the distribution of the 7222 participants by country, indicating a higher participation from Mexico (*n* = 1139) and a lower participation from Turkey and Latvia (*n* = 296 and *n* = 300, respectively). 

**Figure 2:**
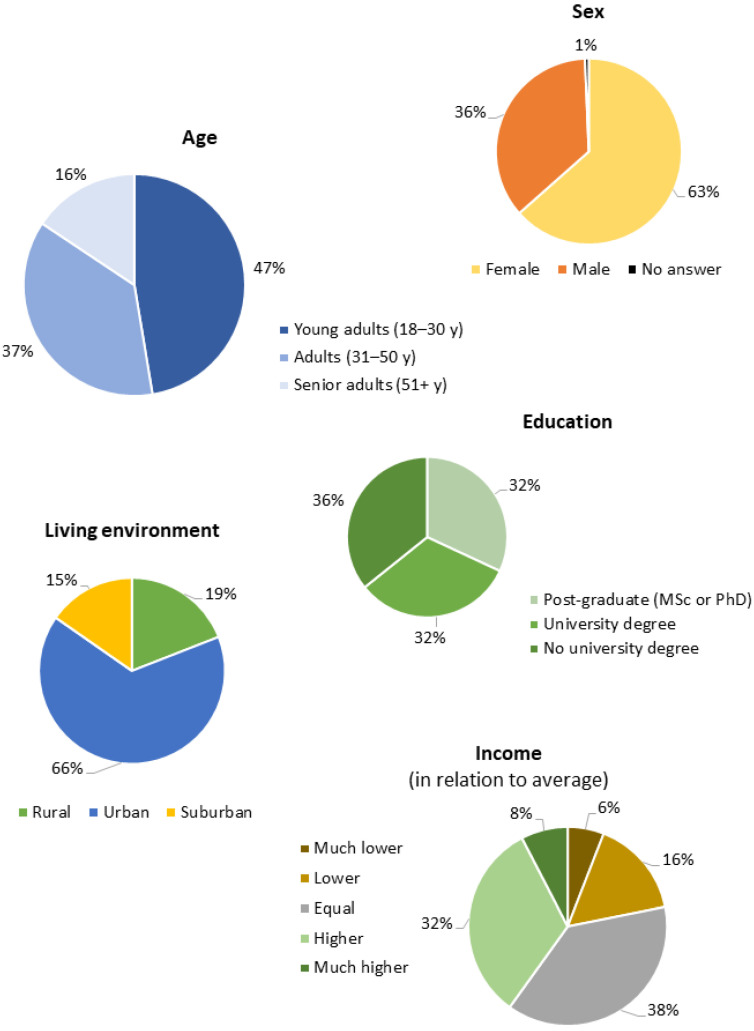
Sociodemographic characteristics of the sample according to age, sex, education, living environment, and income.

Figure 2 shows the distribution of the participants according to age, sex, education, living environment, and income. The majority of the participants were female (63%), and the age class most represented was young adults (aged between 18 and 30 years, which represented 47% of the sample). With respect to education, 36% did not have a university graduation, while 32% had a university degree and 32% had post-graduate studies. A high percentage of participants resided in urban environments (66%), and most participants (38%) had an income equal to the average income in their own countries.

### 3.2. Analysis of the Scale Items with Factor Analysis

Bartlett’s test was significant (*p* < 0.0005), thus leading to the rejection of the null hypothesis “H0: The correlation matrix is equal to the identity matrix”, and, in fact, in the correlation matrix, there were some associations between the variables, with two values above 0.5 (r = 0.588 between V1 and V2, r = 0.502 between V1 and V5) (The list of variables is listed as the items in Appendix A). 

According to the reference values for the KMO [35], the obtained value of 0.836 can be considered as good. All the values of the Measure of Sampling Adequacy (MSA) were higher than 0.5, based on the anti-image matrix, confirming that all the variables could be included in the analysis. The lowest MSA was 0.592 for variable V8, and the highest was 0.894 for variable V13.

The FA with the PCA and Varimax rotation extracted four factors that explained 56.31% of the variance. Practically all communalities were higher than 0.5, except for variables V8 (0.467) and V14 (0.463), so these two variables were found as not suitable to be included in the analysis. For this reason, a second FA was run without variables V8 and V14. 

For the second FA, Bartlet’s test was again significant (*p* < 0.0005) and the value of KMO increased to 0.848, and the values of MSA also increased, resulting in a minimum of 0.712 (for V7) and a maximum of 0.899 (for V13). This solution extracted four factors again, explaining 62.0% of the variance. This percentage indicates that only about 56% of the variance in the data could be explained by this solution, and the rest of the variance is determined to other conditions. In addition, the factor solution was obtained assuming the criteria of getting eigenvalues greater than 1, as indicated in Materials and Methods,, which is usually the criteria for choosing the factors to be included in an analysis. All communalities were higher than 0.5, the highest value being 0.847 (variable V3 has 84.7% of its variance explained by the solution), and the lowest being 0.534 (53.4% VE for variable V10). The results obtained are shown in Table 1, and they include the validation by Cronbach’s alpha (α), measuring the internal consistency of each factor [34]. The distribution of the items by the factors resulted in one factor (F1) that contained five variables related to the economic impact of EIs, another factor (F2) that included four variables related to the motivation for the consumption of EIs, a third factor (F3) that included two variables, both related to the places of purchase of EIs, and a fourth factor (F4) that included only one variable, which was the only item that was given as a false statement. The internal consistency of factors F1 and F2 was good (α = 0.785 and α = 0.702, respectively), while for factor F3, the value of alpha was acceptable (α = 0.560). 

**Figure 3:**
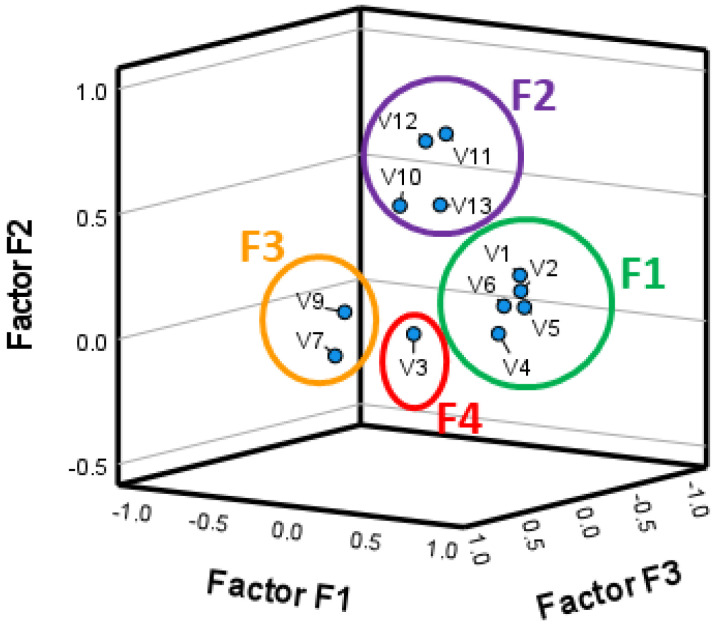
Factor Analysis component plot for solution obtained with Varimax rotation.

Figure 3 presents a graphical representation of the factors in the rotated space, clearly showing the separation of the four factors. Factor F1 was the most relevant, including five variables (V1, V2, V4, and V6), that, together, explain 22.6% of the total variance. Factor F2 explains 17.0% of the total variance and contains four variables (V10, V11, V12, and V13). Factor F3 contains only two variables (V7 and V8) and explains 12.3% of variance, while the last factor, F4, explains 9.5% of the variance and included only one variable (V3). 

### 3.3. Cluster Analysis

Based on the factorial structure obtained, a cluster analysis was performed, allowing for distinguishing three clusters to group the cases. These groups of participants were characterized according to the sociodemographic variables as presented in Table 2. Significant differences at the level of 5% (*p* < 0.05) were observed between the clusters for the three sociodemographic variables considered (sex, age, and education), but the associations were weak, as indicated by the low values of the Cramer’s coefficients. A higher percentage of female participants (42.6%) fit into cluster C1, while male participants were mostly in cluster C2 (38.9%). With respect to age, while cluster C2 had more young adults (40.8%), cluster C1 had more adults (44.1%) and senior adults (44.2%). In terms of education level, cluster C1 contained a higher percentage of the participants with the highest level of instruction (48.5%).

Table 3 presents the results of the cross-tabulation between the cluster membership and the geographical variables, country and living environment. In both cases, the Chi-square test indicated significant differences (*p* < 0.05), although the association was moderate for variable country (V = 0.320), while being low for living environment (V = 0.030). The results reveal that cluster C1 contains more Greek (59.9%), Latvian (66.0%), Lithuanian (45.3%), Polish (62.7%), Portuguese (58.3%), Romanian (47.4%), and Spanish (51.5%) participants, while in cluster C2 predominate the Brazilians (49.7%), Croatians (42.8%), Lebanese (73.7%), Mexicans (41.6%), Serbians (60.2%), Slovenians (42.9%), and Turkish (58.8%) participants. Regarding living environment, also presented in Table 3, the results highlight similar distributions of rural and suburban participants between clusters C1 and C2, while those living in urban environments are clearly more representative in cluster C1 (41.1%).

Table 4 contains the results for the cross-tabulation between income and cluster membership, revealing that clusters C1 and C2 have similar percentages of participants in practically all income categories, except for the high-income participants, who are present in cluster C1 in a higher percentage (42.8%). In this case, the differences were significant *p* (*p* < 0.05), but the association was low (V = 0.048).

### 3.4. Perceptions of the Participants

Table 5 presents the results obtained for each of the items isolated, and they show a high level of agreement towards all items except for items V3, V8, and V14. As previously seen, questions V8 and V14 were not considered to be consistent enough to be part of the scale, and in relation to question V3, this was included, but as an isolated factor. Question V3 corresponds to a false statement, whereas questions V8 and V14 may be strongly dependent on the origin of the participants, since, in the sample, most participants were from non-insect-eating countries, but there were also many participants from countries where Eis are part of the culinary culture, such as, for example, Mexico, in some of the country’s regions.

Items with a very high percentage of agreement or strong agreement included, for example, V7, V10, and V13, dealing with, respectively, difficulty in finding EIs on sale, the knowledge acting as a strong motivator for EI consumption, and the role of personalities and influencers in increasing the will to consume EIs.

**Figure 4:**
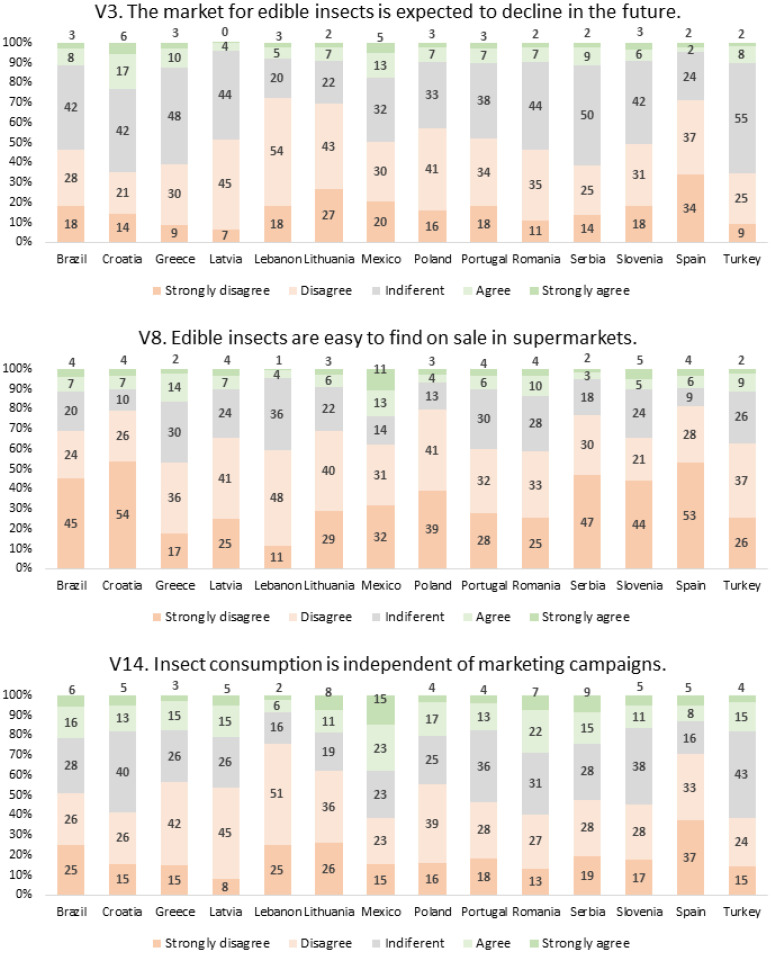
Responses obtained to items V3, V8, and V14, separated by country.

**Figure 5:**
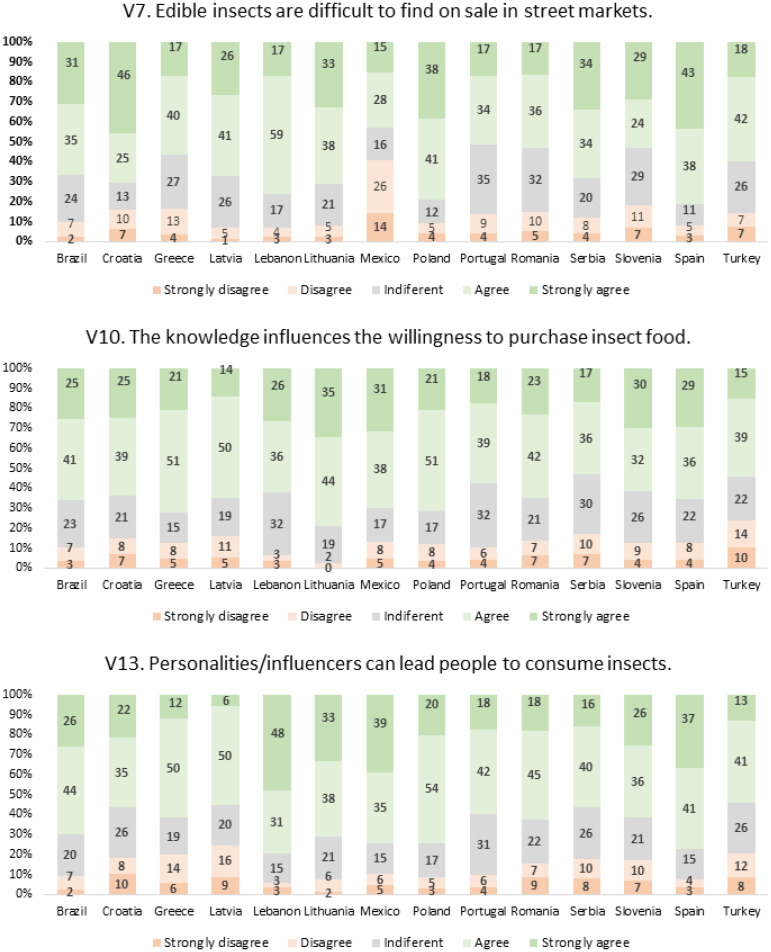
Responses obtained to items V7, V10, and V13, separated by country.

For the items considered problematic, V3, V8, and V14, as well as for those that gathered the highest level of agreement globally, V7, V10, and V13, the responses obtained from the participants from each country are shown in Figure 4 and Figure 5. For the other variables that were not so relevant, similar results are presented in Appendix B, in table format.

Regarding the results in Figure 4, country differences are visible for all three items. For V3, the false item separated by the FA into one factor, F4, the highest percentage of people who did not manifest an opinion was found for Turkey (55% of indifferent score), and the lowest for Lebanon (20%). Since this statement corresponds to a false affirmation, responding disagreement scores corresponded to a correct perception. Therefore, higher percentages of disagreement scores (1 = strongly disagree and 2 = disagree, altogether) were found for Lebanon (18 + 54 = 72%), Spain (24 + 37 = 71%), and Lithuania (27 + 43 = 70%), revealing a predominance of correct perception in these countries. The two other variables considered to be problematic were items V8 and V14, which were excluded from the FA for not being consistent enough. The results in Figure 4 show that, for V8, the only country where a slightly higher percentage of agreement scores was found (4 = agree and 5 = strongly agree, jointly) was Mexico (13 + 11 = 24%), showing that, in some regions of Mexico, the commercialization of insects is a regular practice. Also, the results for item V14 show a similar trend, with a predominance of positive answers for Mexican participants, with 38% (23 + 15 = 38%) believing that consumption of EIs does not depend on marketing campaigns.

In Figure 5, it presents the results obtained for items that gathered the highest level of agreement globally. For item V7, the results show that EIs are generally difficult to find on sale in street markets, particularly in Lebanon (17 + 59 = 79%), Poland (38 + 41 = 79%), and Spain (38 + 43 = 81%). In what concerns the influence of knowledge in the purchase of EIs (item V10), highest agreement was found for Lithuania (35 + 44 = 79%), Greece (21 + 51 = 72%), and Poland (21 + 51 = 72%). Finally, the results for item V13 revealed that, in Lebanon (31 + 48 = 79%) and Spain (41 + 37 = 78%), the highest percentages of agreement were found for the influence of personalities and influencers in encouraging the consumption of EIs.

A mean score was calculated for each participant considering the responses given to all the items, and those values were then tested for differences according to sociodemographic variables, country, age, sex, education, living environment, and income. The results presented in Table 6 show significant differences according to country, sex, education, living environment, and income. On the other hand, age was not found to be associated with the perceptions, since no significant differences were observed between age groups. Nevertheless, in this case, the value of significance was marginal (*p* = 0.053), just slightly above the level of significance considered in the analyses (*p* < 0.05). 

## 4. Discussion

Edible insects may be capable of providing economic benefits, since they seem to be a more sustainable and environmentally friendly nutrient source than other animals and have a high feed-to-meat conversion efficiency [43]. More than two billion people globally routinely consume insects [44]. In 130 countries, 3071 ethnic groups consume over 2086 insect species, with the African, Australian, Asian, and South American continents traditionally being the most entomophagous regions [43,45]. 

The results of our research revealed differences between countries, sex, education, living environment, and income, and therefore, these sociodemographic characteristics were found to significantly influence perceptions about EIs. Different cultures accept the consumption of EIs as food to different degrees. Acceptance can be influenced by complex themes such as regional values, trends in health and sustainability, and cultural attitudes towards food. For example, northern Europeans tend to have a more positive view of insect food than central Europeans [46]. According to a study developed in Italy by Laureati et al. [47], age, gender, awareness of the topic, and food neophobia were found to be the most influential factors in this regard for Italian consumers. The readiness to accept insects was stronger among males than females and was stronger among younger consumers than among older consumers. 

Food and food service industries can tailor their marketing efforts to better meet the specific needs of different consumer segments, taking into account these sociodemographic characteristics, which allows companies to develop strategies to achieve EI consumer development and adapt pricing and promotion [48].

Despite the numerous benefits of EI consumption, consumer acceptance still remains one of the obstacles to their utilization as a protein food source, especially in developed countries where insects are viewed in disgust by the majority of the population [49]

According to Wendin and Nyberg [50], even though sustainability factors are important, they are seldom the main reasons influencing insect consumption. Instead, a complexity of emotional factors, such as disgust and neophobia, as well as familiar tastes, textures, and contexts, were found to have a major influence. In addition, exposure and positive tasting experiences have been identified as important factors for increasing acceptance. 

Our study revealed that knowledge is a strong motivator for the consumption of EIs, so we believe that a strategy should be developed by food manufacturers to educate consumers on the nutritional benefits, changing the negative perceptions (thus increasing consumer willingness to eat insects). Both Mancini et al. [51] and Barton et al. [52] also proposee that education paired with informative tasting sessions could be one strategy to reduce the rejection of and disgust for the use of insects as food. Jones [53] suggested that another strategy could be teaching some subjects in the classroom about sustainable food choices, but also in all areas of the school, for example, in the school canteen.

Huis et al. [44] compiled in their study some strategies to convince customers to consume of EIs: (1) emphasising that insects are nutritionally adequate; (2) incorporating them in an unrecognisable form in familiar products; (3) making insect products delicious; (4) giving people a taste experience; (5) marketing insect-based products by taste; (6) providing detailed information about the insect product, taking into consideration that sustainability may not be the most convincing factor; (7) using celebrities to promote the product; (8) targeting specific groups such as sensation seekers or children; and (9) devising market strategies, such as using stylistic images and choosing supermarkets for retailing. 

According to Ventanas et al. [54], marketing strategies should emphasise positive emotions (i.e., adventurous) to persuade potential consumers. 

Megido et al. [55] observed that people are more willing to consume EIs when presented in other food forms, e.g., cookies, energy bars, burgers, and sandwich spreads, among others, and, according to a study conducted by Meyer-Rochow et al. [56], insects processed into flour or pastes have a greater chance of being accepted when insect images are not displayed on the packaging. 

Our study also revealed that a major concern of consumers was finding EIs on sale in supermarkets and that they are mainly found in specialized shops. In a study conducted in Thailand by Phonthanukitithaworn et al. [57], marketing strategies are crucial and should include attractive advertising and promotion strategies, as well as accessible distribution channels; promotional tools such as buy-one-get-one-free could stimulate insect consumption. In a study conducted by Alemu et al. [58], in Kenya, they concluded that affirmative recommendations are particularly important for processed EIs, and consumers prefer to buy this type of product in kiosks or supermarkets than at local marketplaces. 

Our study also revealed that personalities/influencers can lead people to consume insects. Also, according to a study conducted by Lensvelt and Steenbekkers [59], if the information about Eis is provided by scientists, close relatives, the government, or persons using the product, it may influence EI consumption. Moreover, Park et al. [60] found that males in the USA could be influenced by using advertisements for EI, featuring actors/actresses and athletes, while for females, it was only the first group. 

## 5. Conclusions and Limitations

The results of the scale analysis revealed a structure composed of four factors that explained 62% of the variance, one factor gathering the items related to the economic impact of EIs, another factor including the items related to the motivation for consumption of EIs, a third factor joining together two items related to the places of purchase of EIs, and a fourth factor containing a single item, which was the question presented to the participants as a false statement. The FA excluded two items, one related to the easiness of finding EIs on sale in supermarkets and another about the influence of marketing campaigns on the consumption of EIs. The internal consistency of factors F1 and F2 was found to be good, while for factor F3, it was acceptable. Factor F4 is a single item, therefore, no internal reliability applies to this case. Taking into consideration the obtained results, it was concluded that the questionnaire items’ factorial structure containing four factors was found to be appropriate for assessing the perceptions of consumers about the economic and social impact of EIs in multiple countries.

The results further showed that aspects related to difficulty in finding EIs on sale, knowledge acting as a strong motivator for EI consumption, and the role of personalities and influencers in increasing the will to consume EIs, received by the participants a high level of agreement, indicating strong perceptions about these issues.

Finally, sociodemographic variables like country, sex, education, living environment, and income were found to significantly influence the perceptions of the participants in the study, while age was not found to be significantly related to the perceptions. 

This work, although providing valuable insight, has some limitations that can, to some extent, limit the applicability of the results. One of them is related to the uneven distribution of the participants between countries. While the populations in the different countries are highly variable in size, the number of obtained responses was not directly proportional, and this resulted from the difficulty in recruiting participants to respond to the questionnaire, especially in some of the countries. For example, Brazil is a very large country, and the number of obtained responses was rather low compared to Croatia, a much smaller country. One other limitation was related to the uneven distribution of the participants by the different classes according to the sociodemographic variables considered, namely much more female participants, who usually are more predisposed to answer questionnaires, more participants living in urban environments, a high percentage of post-graduate participants, and a high representativeness also of young adults up to 30 years. These dissimilarities can introduce some bias into the interpretation of the results.

## Figures and Tables

**Table 1:** The final solution obtained through factor analysis.

Factor	%VE ^1^	Item	Loading	Alpha ^2^
F1	22.6%	V1. Insect production can contribute to increase the income of families in low-income areas	0.692	0.785
		V2. Insects provide protein foods at cheap prices	0.694	
		V4. Presently, the Asia–Pacific and Latin America areas account for more than half of the edible insects’ market	0.692	
		V5. In some countries insect farming is becoming a key factor to fight against rural poverty	0.774	
		V6. The income generated from insects can be affected by market fluctuations in price derived from availability	0.677	
F2	17.0%	V10. The level of knowledge influences the willingness to purchase insect food	0.557	0.702
		V11. Price is among the motivations to consume insect foods	0.789	
		V12. The consumption of insects and derived foods depends on availability	0.777	
		V13. Personalities/influencers can lead people to consume insects	0.547	
F3	12.3%	V7. Edible insects are difficult to find on sale in street markets	0.820	0.560
		V8. Edible insects are on sale only in specialized shops	0.777	
F4	9.5%	V3. The market for edible insects is expected to decline in the future	0.920	N.A. ^3^

^1^ VE = Variance explained. ^2^ Cronbach’s alpha. ^3^ N.A. = not applicable, single item in the factor.

**Table 2:** Association between cluster membership and sociodemographic variables.

Variables	Groups	Cluster C1 (%)	Cluster C2 (%)	Cluster C3 (%)	Total (%)
Sex (*p* ^1^ < 0.001; V ^2^ = 0.038)	Female	42.6	36.2	21.2	100.0
	Male	37.4	38.9	23.8	100.0
	No answer	31.3	45.8	22.9	100.0
Age group (*p* ^1^ < 0.001; V ^2^ = 0.058)	Young adults (18–30 y)	36.5	40.8	22.6	100.0
	Adults (31–50 y)	44.1	34.5	21.4	100.0
	Senior adults (51 y or over)	44.2	33.2	22.5	100.0
Education level (*p* ^1^ < 0.001; V ^2^ = 0.078)	Post graduate (MSc ot PhD)	48.5	32.3	19.2	100.0
	University degree	36.6	40.3	23.0	100.0
	No University degree	37.2	38.8	24.0	100.0

^1^ Significance of the Chi-square test. ^2^ Cramer’s coefficient.

**Table 3:** Association between cluster membership and demographic variables.

Variables	Groups	Cluster C1 (%)	Cluster C2 (%)	Cluster C3 (%)	Total (%)
Country (*p* ^1^ < 0.001; V ^2^ = 0.320)	Brazil	37.9	49.7	12.4	100.0
	Croatia	24.2	42.8	33.0	100.0
	Greece	59.9	4.4	35.7	100.0
	Latvia	66.0	15.3	18.7	100.0
	Lebanon	22.4	73.7	3.9	100.0
	Lithuania	45.3	41.0	13.7	100.0
	Mexico	26.6	41.6	31.8	100.0
	Poland	62.7	13.8	23.5	100.0
	Portugal	58.3	38.5	3.2	100.0
	Romania	47.4	45.3	7.3	100.0
	Serbia	11.9	60.2	27.9	100.0
	Slovenia	27.3	42.9	29.8	100.0
	Spain	51.5	20.0	28.5	100.0
	Turkey	36.5	58.8	4.7	100.0
Living environment (*p* ^1^ = 0.012; V ^2^ = 0.030)	Rural	40.3	40.4	19.3	100.0
	Urban	41.1	36.3	22.5	100.0
	Suburban	38.7	37.4	23.9	100.0

^1^ Significance of the Chi-square test. ^2^ Cramer’s coefficient.

**Table 4:** Association between cluster membership and economic variables.

Variables	Groups	Cluster C1 (%)	Cluster C2 (%)	Cluster C3 (%)	Total (%)
Income (*p* ^1^ < 0.001; V ^2^ = 0.048)	Much lower than average	37.0	35.8	27.1	100.0
	Lower than average	38.2	37.0	24.8	100.0
	Equal to average	39.8	37.7	22.5	100.0
	Higher than average	42.8	35.8	21.5	100.0
	Much higher than average	42.9	41.9	15.2	100.0

^1^ Significance of the Chi-square test. ^2^ Cramer’s coefficient.

**Table 5:** Scores attributed for each item, considering the global sample.

Variable	Items	% of Answers				
		Level of Agreement ^1^				
		1	2	3	4	5
V1	Insect production can contribute to increase the income of families in low-income areas	5.7	9.9	32.3	37.4	14.7
V2	Insects provide protein foods at cheap prices	5.3	11.2	33.7	34.3	15.4
V3	The market for edible insects is expected to decline in the future	17.5	33.4	37.2	8.6	3.3
V4	Presently, the Asia–Pacific and Latin America areas account for more than half of the edible insects’ market	2.9	5.3	42.1	35.8	13.8
V5	In some countries insect farming is becoming a key factor to fight against rural poverty	3.5	6.7	46.8	33.0	9.9
V6	The income generated from insects can be affected by market fluctuations in price derived from availability	3.5	7.3	47.8	31.3	10.2
V7	Edible insects are difficult to find on sale in street markets	5.9	10.9	21.2	35.2	26.9
V8	Edible insects are easy to find on sale in supermarkets	34.5	32.6	20.5	8.0	4.4
V9	Edible insects are on sale only in specialized shops	4.6	8.9	28.9	39.6	18.0
V10	Level of knowledge influences the willingness to purchase insect food	4.8	7.6	21.7	41.0	24.9
V11	Price is among the motivations to consume insect foods	10.4	17.7	33.8	27.6	10.4
V12	The consumption of insects and derived foods depends on availability	6.2	14.4	28.4	37.4	13.6
V13	Personalities/influencers can lead people to consume insects	5.5	7.7	20.5	40.9	25.4
V14	Insect consumption is independent of marketing campaigns	18.9	31.5	27.9	15.1	6.6

^1^ Values on a scale from 1 to 5, for which: 1 = strongly disagree, 2 = disagree, 3 = no opinion, 4 = agree, and 5 = strongly agree.

**Table 6:** Perceptions according to sociodemographic variables.

Variables	Groups	Perceptions Mean ± s.d. ^1^	Significance ^2^
Country	Brazil	3.24 ± 0.44 ^bcd^	*p* < 0.001
	Croatia	3.16 ± 0.50 ^abc^	
	Greece	3.18 ± 0.49 ^abc^	
	Latvia	3.12 ± 0.31 ^a^	
	Lebanon	3.47 ± 0.49 ^f^	
	Lithuania	3.26 ± 0.33 ^cd^	
	Mexico	3.42 ± 0.5 ^ef^	
	Poland	3.31 ± 0.39 ^de^	
	Portugal	3.18 ± 0.49 ^abc^	
	Romania	3.23 ± 0.51 ^bcd^	
	Serbia	3.13 ± 0.57 ^ab^	
	Slovenia	3.22 ± 0.48 ^abcd^	
	Spain	3.25 ± 0.43 ^cd^	
	Turkey	3.14 ± 0.55 ^ab^	
Sex	Female	3.24 ± 0.48 ^a^	*p* = 0.014
	Male	3.28 ± 0.50 ^ab^	
	No answer	3.31 ± 0.57 ^b^	
Age class	Young adults (18–30 y)	3.26 ± 0.50 ^a^	*p* = 0.053
	Adults (31–50 y)	3.26 ± 0.47 ^a^	
	Senior adults (51+ y)	3.22 ± 0.47 ^a^	
Education	Post-graduate (MSc or PhD)	3.25 ± 0.44 ^ab^	*p* < 0.001
	Completed university degree	3.28 ± 0.49 ^b^	
	No university degree	3.23 ± 0.52 ^a^	
Living environment	Rural	3.22 ± 0.51 ^a^	*p* = 0.007
	Urban	3.26 ± 0.49 ^ab^	
	Suburban	3.28 ± 0.46 ^b^	
Household income in relation to average	Much lower	3.25 ± 0.58 ^ab^	*p* = 0.009
	Lower	3.28 ± 0.49 ^b^	
	Equal	3.23 ± 0.49 ^a^	
	Higher	3.27 ± 0.45 ^b^	
	Much higher	3.26 ± 0.51 ^ab^	
Global sample		3.25 ± 0.49	

^1^ Values on a scale from 1 to 5, for which: 1 = strongly disagree, 2 = disagree, 3 = no opinion, 4 = agree, and 5 = strongly agree. ^2^ ANOVA with post hoc test of Tukey at a level of significance of 5% (*p* < 0.05). Mean values with different superscripts are significantly different.

## Data Availability

Data may be requested from the corresponding author.

## Appendix A

The items of the questionnaire used to investigate the Economic and Social aspects (items 1 to 6) as well as the Commercialization and Marketing aspects of EIs (items 7 to 14) are shown in Table A1.

**Table A1:** List of items in the questionnaire (Scale: 1 = Strongly disagree; 2 = Disagree; 3 = No opinion; 4 = Agree; and 5 = Strongly agree).

Variable	Items	Level of Agreement				
		1	2	3	4	5
1	Insect production can contribute to increase the income of families in low-income areas					
2	Insects provide protein foods at cheap prices					
3	The market for edible insects is expected to decline in the future					
4	Presently, the Asia–Pacific and Latin America areas account for more than half of the edible insects’ market					
5	In some countries insect farming is becoming a key factor to fight against rural poverty					
6	The income generated from insects can be affected by market fluctuations in price derived from availability					
7	Edible insects are difficult to find on sale in street markets					
8	Edible insects are easy to find on sale in supermarkets					
9	Edible insects are on sale only in specialized shops					
10	The level of knowledge influences the willingness to purchase insect food					
11	Price is among the motivations to consume insect foods					
12	The consumption of insects and derived foods depends on availability					
13	Personalities/influencers can lead people to consume insects					
14	Insect consumption is independent of marketing campaigns					

## Appendix B

The results of the percentages of the scale scores obtained for variables V1, V2, V4, V5, V6, V9, V11, and V12, according to country are shown in Table A2.

**Table A2:** Percentage of participants’ responses according to country, for the less relevant variables.

Variable ^1^	Score ^2^	Percentage of Responses													
		Country ^3^													
		BR	HR	GR	LV	LB	LT	MX	PL	PT	RO	RS	SI	ES	TR
V1	1	4.3	12.1	6.8	5.3	3.1	7.5	3.1	3.5	4.7	6.3	7.3	4.8	5.0	7.4
	2	7.8	15.3	11.2	19.7	3.1	14.5	5.8	5.0	6.5	10.0	13.1	8.7	7.8	20.6
	3	37.0	32.7	37.3	44.0	19.3	29.0	17.9	31.3	39.1	33.9	41.0	40.4	36.9	32.8
	4	35.1	29.9	40.3	27.7	54.9	33.3	39.0	48.3	39.1	43.5	31.7	31.5	34.1	33.1
	5	15.8	10.1	4.6	3.3	19.6	15.7	34.2	11.9	10.6	6.3	7.0	14.5	16.2	6.1
V2	1	2.8	12.0	4.6	4.7	2.8	8.2	3.4	2.5	4.6	5.9	3.8	3.9	6.4	7.8
	2	8.1	18.7	11.0	13.3	2.8	19.4	11.5	6.7	8.0	10.4	9.3	9.9	10.8	10.1
	3	41.6	43.1	42.3	39.0	13.7	29.6	19.8	25.8	41.4	40.7	49.1	40.6	29.4	32.4
	4	32.0	20.8	35.4	40.0	47.6	28.4	34.2	50.2	35.1	37.4	28.5	28.0	32.9	41.6
	5	15.5	5.4	6.8	3.0	33.1	14.3	31.2	14.8	11.0	5.7	9.3	17.6	20.5	8.1
V4	1	1.9	5.8	2.4	1.0	2.0	6.5	2.2	1.3	2.3	3.5	3.8	1.4	1.7	3.7
	2	9.6	7.9	4.6	2.7	2.5	11.8	5.1	2.5	4.2	3.5	7.6	4.4	4.0	4.1
	3	54.0	42.2	38.8	51.0	29.1	38.4	39.9	43.3	49.9	47.8	44.2	49.9	26.3	47.6
	4	28.6	28.9	45.1	41.7	48.5	30.2	33.0	42.7	34.9	37.8	32.8	28.0	40.5	33.8
	5	5.9	15.2	9.1	3.7	17.9	13.1	19.8	10.2	8.7	7.5	11.6	16.2	27.5	10.8
V5	1	1.9	6.3	3.9	1.3	2.2	5.5	3.2	1.9	4.4	3.3	5.2	1.5	3.1	4.1
	2	6.5	11.4	5.0	5.7	3.1	15.3	5.3	2.7	5.5	6.9	9.6	6.4	6.3	3.7
	3	54.0	42.9	48.9	68.0	36.7	39.0	41.7	53.7	49.1	41.1	50.9	50.9	45.6	51.7
	4	29.2	33.1	37.9	23.7	39.8	28.0	33.5	35.0	33.0	40.2	27.9	28.6	31.5	34.5
	5	8.4	6.4	4.2	1.3	18.2	12.2	16.3	6.7	8.0	8.5	6.4	12.6	13.6	6.1
V6	1	1.2	6.9	2.8	2.7	2.2	5.3	3.1	1.9	3.0	4.5	5.2	2.1	2.6	4.4
	2	10.6	8.6	8.5	6.0	3.9	12.9	7.1	2.5	5.1	7.5	7.8	7.0	7.3	5.7
	3	57.5	54.5	47.5	60.0	37.3	29.2	34.9	57.5	57.1	48.4	55.2	53.4	43.5	58.8
	4	23.6	23.9	35.5	31.0	37.8	32.2	36.9	33.1	28.5	35.4	24.4	26.3	32.5	26.4
	5	7.1	6.1	5.7	0.3	18.8	20.4	18.0	5.0	6.3	4.3	7.3	11.2	14.1	4.7
V9	1	3.4	4.7	3.5	1.0	2.5	2.5	10.2	4.0	3.4	3.5	6.1	3.3	3.1	5.4
	2	6.5	5.1	11.2	3.7	2.5	3.3	19.1	5.8	7.2	5.5	12.2	7.7	9.7	8.4
	3	35.7	23.0	23.9	43.7	23.8	25.3	18.2	29.0	43.3	33.5	34.6	37.3	24.0	39.2
	4	36.0	38.0	50.5	42.7	54.9	34.5	36.1	49.2	36.8	41.5	30.8	32.7	38.4	33.4
	5	18.3	29.2	11.0	9.0	16.2	34.3	16.4	11.9	9.3	16.1	16.3	19.0	24.7	13.5
V11	1	10.6	14.7	12.3	10.0	4.2	16.1	7.0	5.4	6.6	9.6	9.9	13.0	15.8	10.8
	2	12.4	16.5	27.2	31.7	3.6	28.2	14.6	15.4	14.0	16.7	19.5	16.8	16.2	18.2
	3	44.1	34.8	25.8	31.3	31.1	29.8	23.6	37.3	52.9	38.2	37.8	33.1	39.8	26.4
	4	25.8	24.2	28.1	25.0	41.7	16.7	34.9	34.0	20.9	28.5	25.6	25.9	19.3	34.1
	5	7.1	9.8	6.6	2.0	19.3	9.2	19.9	7.9	5.5	7.1	7.3	11.2	8.9	10.5
V12	1	7.5	8.7	9.0	5.7	2.8	5.7	3.2	4.2	6.8	5.9	7.0	7.9	6.3	8.8
	2	12.7	13.8	25.6	20.0	7.0	12.0	9.7	15.6	13.7	12.0	14.0	15.3	16.9	16.2
	3	35.4	31.3	32.9	22.7	27.2	19.0	18.0	20.2	40.4	29.9	35.8	36.4	30.4	32.1
	4	33.2	34.5	28.8	46.3	46.2	39.2	42.3	52.1	32.8	41.7	32.6	29.2	30.8	34.5
	5	11.2	11.5	3.8	5.3	16.8	24.1	26.8	7.9	6.3	10.6	10.8	11.2	15.7	8.4

^1^ V1 = Insect production can contribute to increase the income of families in low-income areas, V2 = Insects provide protein foods at cheap prices, V4 = Presently, the Asia–Pacific and Latin America areas account for more than half of the edible insects’ market, V5 = In some countries insect farming is becoming a key factor to fight against rural poverty, V6 = The income generated from insects can be affected by market fluctuations in price derived from availability, V9 = Edible insects are on sale only in specialized shops, V11 = Price is among the motivations to consume insect foods, and V12 = The consumption of insects and derived foods depends on availability. ^2^ Scale: 1 = Strongly disagree; 2 = Disagree; 3 = No opinion; 4 = Agree; and 5 = Strongly agree. ^3^ BR = Brazil, HR = Croatia, GR = Greece, LV = Latvia, LB = Lebanon, LT = Lithuania, MX = Mexico, PL = Poland, PT = Portugal, RO = Romania, RS = Serbia, SI = Slovenia, ES = Spain, and TR = Turkey.
